# Biomechanical Characteristics of Compensatory Reactive Step Responding to the Simulated Trip Perturbation While Walking in Community‐Dwelling People With Stroke: A Cross‐Sectional Study

**DOI:** 10.1002/hsr2.71727

**Published:** 2026-02-26

**Authors:** Suk‐Ping Chan, Patrick Wai‐Hang Kwong, Gladys Lai‐Ying Cheing, Sharon Man‐Ha Tsang

**Affiliations:** ^1^ Department of Rehabilitation Sciences The Hong Kong Polytechnic University The Hong Kong Special Administrative Region China

**Keywords:** biomechanical characteristics, one‐dimensional statistical parametric mapping, reactive step, simulated trip perturbation, stroke

## Abstract

**Background and Aims:**

Falls pose a significant public health risk for community‐dwelling stroke survivors. Current research on biomechanical parameters to trip perturbation in standing tasks often fails to predict fall risk or balance recovery during dynamic tasks such as walking. Moreover, phase‐specific biomechanical adaptations during recovery from perturbations remain underexplored. One‐dimensional statistical parametric mapping (SPM1D) was used in the current research for analyzing time‐series biomechanical data and to investigate joint angular profiles during reactive stepping following trip‐like gait perturbations. The aim was to compare these biomechanical characteristics between stroke survivors and healthy controls.

**Methods:**

Fourteen participants with stroke and ten healthy controls were assessed using a 16‐camera motion capture system. Participants walked at self‐selected usual walking speeds on a split‐belt treadmill and underwent a simulated trip perturbation, triggered by backward treadmill acceleration during initial foot contact. Biomechanical variables at the reactive step touchdown were derived using the Vicon plug‐in‐gait full‐body model. Biomechanical characteristics (joint angles and moments) showing significant between‐group differences at reactive step touchdown were initially identified, and SPM1D was then utilized to analyze joint angles and moments across three equal phases of the reactive step cycle (initial, middle, and end). Independent‐samples *t*‐tests complemented SPM1D were used to identify between‐group differences, with significance set at *p* ≤ 0.05.

**Results:**

Compared to controls, stroke participants showed increased trunk flexion, knee flexion, and ankle dorsiflexion angles on the perturbed side, along with decreased ankle dorsiflexion moment and altered upper limb movement strategies. SPM1D revealed phase‐specific differences that increased shoulder abduction during the initial phase on the reactive step side, increased shoulder external rotation in the middle phase on the perturbed side, and greater trunk flexion and ankle dorsiflexion angle but reduced ankle dorsiflexion moment on the perturbed side during the end phase.

**Conclusion:**

Participants with stroke exhibit distinct, phase‐dependent biomechanical adaptations during reactive stepping post‐trip perturbation.

## 1 Introduction

### 1.1 Background

Falls constitute a major global health concern and rank as the second leading cause of unintentional injury‐related deaths worldwide. Each year, millions of people experience falls, many resulting in serious injuries that require medical intervention [1]. Stroke survivors, in particular, are at elevated risk for falls, which frequently lead to increased mortality, morbidity, and long‐term disability [2]. Among community‐dwelling stroke survivors, those with impaired balance and mobility face an even greater likelihood of falling [3]. Reactive stepping, the ability to quickly place the foot in response to an external perturbation to prevent a fall, is often compromised in ambulatory stroke individuals [4]. In response to a larger external perturbation, stroke survivors' reactive stepping responses tend to be impaired, reducing their ability to regain balance effectively [5].

Previous investigations have provided valuable insights into biomechanical factors associated with fall risk in stroke survivors [5, 6, 7]. However, many early studies focused on biomechanical parameters during trip perturbations in standing tasks, limiting their clinical relevance for dynamic, functional tasks such as walking under trip conditions. Furthermore, there is a paucity of research examining phase‐specific biomechanical characteristics throughout reactive recovery responses to gait perturbations.

To address these gaps, advanced analytical methods like one‐dimensional statistical parametric mapping (SPM1D) can be employed to analyze joint angular time series data collected during reactive stepping of recovery response after trip‐like gait perturbations. SPM1D, grounded in Random Field Theory [8, 9], enables comprehensive statistical analyses of entire biomechanical trajectories rather than isolated time points [10]. This increases statistical power while reducing false positives relative to zero‐dimensional approaches [11]. SPM1D has been successfully applied in analyzing movement patterns among both healthy individuals and those with neurological impairments such as stroke [12]. The importance of analyzing the time‐dependent response in reactive stepping lies in understanding how response recovery unfolds dynamically over time after a perturbation, which is critical for fall prevention and rehabilitation. Moreover, using a split‐belt treadmill (SBT) allows precise control over gait perturbations during walking, facilitating realistic simulations of trip events. Understanding the time‐dependent, phase‐specific biomechanical responses during reactive stepping is critical for developing targeted interventions for fall prevention and rehabilitation in stroke populations.

This study aimed to compare the biomechanical characteristics of reactive stepping during simulated trip perturbations while walking between community‐dwelling stroke participants and healthy controls. It also sought to examine differences in joint angular time profiles across these groups. The hypotheses were that statistically significant differences would be observed in (1) the overall biomechanical characteristics of reactive stepping and (2) the joint angular profiles during reactive stepping, comparing stroke participants with healthy controls. Specifically, SPM1D analysis was expected to reveal distinct difference‐in‐difference trends throughout the entire movement continuum, reflecting altered motor control mechanisms post‐stroke. SPM1D was applied here as a secondary, exploratory analysis to examine difference‐in‐difference trends across the movement continuum and to provide pilot findings that would inform sample‐size and hypothesis development in future confirmatory studies.

## 2 Methods

### 2.1 Study Design

This cross‐sectional study was approved by the Research Ethics Committee (Kowloon Central/Kowloon East) and the Institutional Review Board of The Hong Kong Polytechnic University (Reference No. HSEARS20210916001). All participants received detailed information about the study's objectives, procedures, potential benefits, and risks, and provided written informed consent prior to participation.

**Figure 1:**
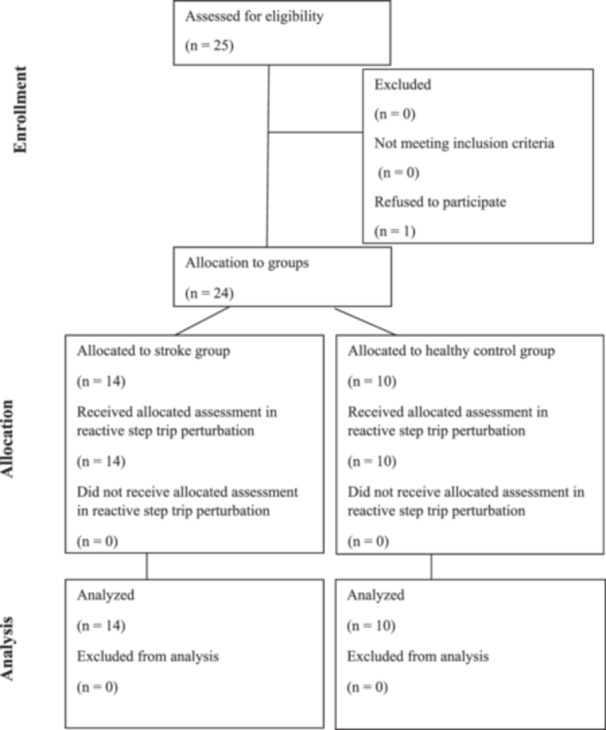
Flowchart of participants.

Fourteen community‐dwelling (living independently, not in nursing homes) stroke participants aged 18 to under 60 years and 10 healthy control were recruited. Younger stroke participants were selected due to their greater social activity and evidence suggesting that younger age predicts better gait speed recovery across stroke phases [13]. Stroke participants were independently ambulatory with Modified Functional Ambulation Classification (MFAC) Category VI or VII, indicating indoor or outdoor independent ambulation [14, 15]. Exclusion criteria applied for stroke participants were the presence of coexisting neurological disorders, significant cardiac or orthopedic conditions, cognitive or visual impairments unrelated to stroke that could influence balance or gait, inability to comply with instructions, or a history of falls preceding the stroke event. For healthy controls, the exclusion criteria were consistent with those for stroke participants, except for the requirement of a confirmed medical diagnosis of stroke in the stroke group. Figure 1 outlines participant flow. Demographics were extracted from hospital records, and the dominant limb in controls was determined by asking which foot they would use to kick a ball.

Sample size was calculated post‐initial data collection using G*Power for the Independent‐samples *t*‐test, with a type I error rate of 5% and a power of 80%. Based on trunk flexion angle effect size, a minimum of five participants per group was required. A conservative screening led to recruitment of 25 individuals; one stroke patient declined participation.

Spinal and limb biomechanical parameters (joint angles and moments) during reactive stepping in response to a simulated trip perturbation were obtained using a 16‐camera Vicon Nexus 2 motion capture system (Vicon Motion Systems Ltd., USA) with 39 passive reflective markers placed at standard anatomical landmarks. Passive reflective markers were positioned by a trained assistant specializing in gait laboratory procedures, adhering to standardized placement protocols to ensure data accuracy and reliability. The Vicon plug‐in‐gait full‐body model (version Nexus 2) calculated biomechanical variables. The trip simulation was conducted on a split‐belt treadmill (Bertec Split‐belt Treadmill, USA) with side‐split dual belts. Three reflective markers affixed to the trip‐triggering belt allowed precise detection of trip onset via sudden marker acceleration. The definition of trip and reactive step parameter relating to the determination of the biomechanical characteristics was indicated in Table 1.

**Table 1:** Definition of trip and reactive step parameters for biomechanical analysis.

Trip and reactive step parameter	Definition
Self‐selected usual walking speed	The individual usual walking speed chosen naturally by each participant while walking on the split‐belt treadmill (SBT) prior to perturbation.
Trip side	The paralyzed (affected) side for stroke participants and the nondominant side for healthy controls, where the trip perturbation is applied.
Reactive step side	The nonparalyzed (nonaffected) side for stroke participants and the dominant side for healthy controls, representing the limb used for the compensatory reactive step.
Trip protocol	The perturbation testing trial involved a sudden backward treadmill belt acceleration with an intensity set at 0.77 m/s above the participant's self‐selected usual walking speed, achieving an acceleration of 19.24 m/s^2^ [7]. This perturbation was triggered while participants walked continuously at their self‐selected usual walking speed on the SBT.
Time point of trip	The exact moment marking the onset of the trip, identified by a sudden increase in speed of the reflective marker on the treadmill belt under the trip side, indicating the transition from usual walking speed to perturbation.
Reactive step touchdown	The instance of initial foot contact corresponding to the first reactive step taken to regain balance following the perturbation.
Reactive step cycle	The time interval from the trip onset “time point of trip” to the reactive step touchdown.
Phases of reactive step recovery	The reactive step cycle is divided evenly into three phases: Initial phase (the first third of the cycle), middle phase (the second third), and end phase (the final third of the reactive step cycle).
Joint angle at reactive step touchdown	The angular position of joints (trunk, upper limbs, lower limbs) measured at the moment of reactive step touchdown of the first reactive step.
Joint moment at reactive step touchdown	The joint moment values (normalized by body weight) for trunk, upper limbs, and lower limbs at the reactive step touchdown of the first reactive step.
Reactive step length	The linear distance traveled by the base of support during the reactive step cycle, represented by displacement of the second toe reflective marker.
Reactive step excursion time	The duration of the reactive step cycle, including the time elapsed from trip onset to reactive step touchdown.
Reactive step speed	The speed of reactive stepping, calculated as the reactive step length divided by the reactive step excursion time.
Reactive step time cycle	The reactive step cycle (trip onset to reactive step touchdown) defines 100% of the time interval, which is then segmented equally into the initial, middle, and end phases.

Participants first completed a 5‐min familiarization walking period on the treadmill, during which their self‐selected usual walking speed was identified by gradually adjusting treadmill speed from slow to natural usual walking speed pace. This speed served as individualized baseline for perturbations. The trip simulation involved a backward acceleration of the treadmill belt on the trip‐triggering side during reactive step touchdown: on the paralyzed (affected) side for stroke participants and the nondominant side for controls.

The perturbation protocol was based on the acceleration of the treadmill belt as described in previous research [7], applied relative to each individual's self‐selected walking speed in the current study. A warm‐up trial at usual walking speed plus 0.67 m/s, with acceleration 16.75 m/s^2^, was performed once. Following this, each participant completed a single true perturbation trial at a slightly increased intensity—set at usual walking speed plus 0.77 m/s, acceleration 19.24 m/s^2^.

Only data from the true perturbation trial were analyzed to minimize adaptation effects. Timing of the perturbation was randomized between 10 and 20 steps after starting the familiarization period. An assistant controlled perturbation onset via a control panel. The treadmill belts were stopped manually via the control panel by the assistant after initial contact of the nonaffected limb (stroke) or the dominant limb (controls) of the first reactive step.

The perturbation duration was defined from trip onset to reactive step touchdown. An overhead safety harness was worn for fall protection. Participants rested 1 min between warm‐up and testing. Biomechanical data during reactive stepping were recorded throughout.

Full‐body biomechanical data (joint angles and moments) were recorded during the reactive stepping task. Spatiotemporal parameters, participants' self‐selected usual walking speed, reactive step length, reactive step excursion time, and reactive step speed were also measured. Independent‐samples *t*‐tests (IBM SPSS Statistics Version 28, IBM Corp., Armonk, NY) compared biomechanical responses between stroke and control groups at reactive step touchdown, with significance set at 0.05 (two‐sided). Significant between‐group differences were identified for further analysis. Effect sizes were calculated to quantify the magnitude of group differences. Cohen's d was used for continuous outcomes and interpreted according to conventional thresholds: small (Cohen's *d* = 0.1), medium (Cohen's *d* = 0.4), and large (Cohen's *d* = 0.8) effects [16].

SPM1D in Python [17] was used to analyze the full joint angular time profiles across the entire reactive step cycle, divided into initial, middle, and end phases. SPM1D 2‐sample *t‐*tests tested for differences in mean time series between groups. Statistical significance was determined when the *t* value surpassed the critical test statistical value (*α*) at a significance level of *p* less than or equal to 0.05. SPM(*t*) maps identified the phases within the reactive step cycle where significant biomechanical differences occurred.

## 3 Results

A total of 25 individuals were screened for eligibility. Fourteen participants with stroke (eight females and six males) were allocated to the stroke group, and 10 healthy controls (six females and four males) comprised the control group. One stroke patient declined participation (Figure 1). Among stroke participants, six had left‐side paresis, and eight had right‐side paresis. The groups of stroke and healthy control were comparable in comparable age (stroke 53.07 ± 9.00 vs. control 46.50 ± 18.22, *p* = 0.25), height (stroke 1.62 ± 0.08 m vs. control 1.67 ± 0.08 m, *p* = 0.12) and body mass (stroke 64.34 ± 16.61 kg vs. control 60.99 ± 8.32 kg, *p* = 0.57) (Table 2). The average post‐stroke duration was 45.29 ± 73.05 months, with lesion locations distributed across intracranial (14%), Basal Ganglia (14%), Thalamic (14%), and other or nonspecific areas (58%). Both groups were classified as MFAC Category VII, indicating outdoor independent ambulation.

**Table 2:** Descriptive characteristics and outcomes of stroke and healthy control groups.

Subject	Participant with stroke (*n* = 14)	Healthy controls (*n* = 10)	The Independent‐samples *t*‐test significance *p*‐values	Cohen's *d*
Female/Male (*n*)	8/6	6/4		
Age (years)	53.07 (9.00)	46.50 (18.22)	0.25	0.49
Height (m)	1.62 (0.08)	1.67 (0.08)	0.12	0.66
Body mass (kg)	64.34 (16.61)	60.99 (8.32)	0.57	0.24
Chronicity (months)	45.29 (73.05)	NA	NA	NA
Affected side left/right	6/8	NA	NA	NA
Self‐selected usual walking speed (m/s)	0.27 (0.10)	0.72 (0.16)	< 0.001*	3.43

*Note:* Values were means (standard deviations).

Abbreviation: NA, not applicable.

* *p* < 0.001, significantly different between groups.

Stroke participants selected significantly slower usual walking speeds on the split‐belt treadmill (0.27 ± 0.10 m/s, range: 0.10–0.40 m/s) compared to controls (0.72 ± 0.16 m/s, range: 0.50–0.95 m/s, *p* < 0.001). During the reactive step, stroke participants exhibited markedly slower reactive step speeds (0.62 ± 0.26 m/s) than controls (1.26 ± 0.47 m/s, *p* < 0.001), with controls performing reactive steps over twice as fast. The reactive step excursion time was also significantly longer in stroke participants (0.49 ± 0.25 s vs. 0.29 ± 0.08 s, *p* = 0.02). Stroke participants exhibited significantly slower reactive step speeds compared to healthy controls. The reactive step excursion time was also significantly longer in the stroke group versus controls, reflecting delayed step execution during balance recovery.

The trunk flexion angle of participants with stroke was greater than control at reactive step touchdown (stroke 16.40° ± 9.36° vs. healthy control 3.43° ± 2.48°, *p* < 0.001). At reactive step touchdown, stroke participants demonstrated greater trunk flexion angles than controls, highlighting altered postural control. The knee flexion angle over the trip side at reactive step touchdown was also greater in participants with stroke (stroke 30.38° ± 16.27° vs. healthy control 18.00° ± 10.36°, *p* = 0.05). The results revealed that during perturbation, the knee of the supporting lower limb in stroke patients buckled, providing only limited support to the body during the reactive step, compared to healthy controls. The ankle dorsiflexion angle over the trip side at reactive step touchdown was greater in the stroke group (stroke 14.41° ± 6.11° vs. healthy control 7.49° ± 5.47°, *p* = 0.01), indicating compensatory limb positioning. The ankle dorsiflexion moment over the trip side limb normalized by body weight was smaller in the stroke group (stroke 0.27 ± 0.27 Nm/kg vs. healthy control 0.68 ± 0.37 Nm/kg, *p* < 0.001). The ankle dorsiflexion moment normalized by body weight was significantly reduced in the stroke group compared to controls, suggesting impaired force generation necessary for supporting the step recovery. Shoulder abduction angle over the reactive step side significantly different between groups at reactive step touchdown (stroke 25.22° ± 10.54° vs. healthy control 14.35° ± 6.39°, *p* = 0.01). Shoulder external rotation angle over the trip side at reactive step touchdown (stroke 33.09° ± 19.94° vs. healthy control 14.10° ± 10.78°, *p* = 0.01) was greater in participants with stroke (Table 3). Shoulder abduction angle on the reactive step side and shoulder external rotation angle over the trip side were significantly greater in stroke participants versus controls, reflecting compensatory arm movements during the reactive step.

**Table 3:** Mean values and standard deviations of reactive step biomechanical characteristics of stroke and healthy control groups following simulated trip perturbations on a split‐belt treadmill.

Subject	Participant with stroke (*n* = 14)	Healthy controls (*n* = 10)	The independent‐samples *t*‐test significance *p*‐values	Cohen's *d*
Trunk flexion angle (degree) at reactive step touchdown	16.40 (9.36)	3.43 (2.48)	< 0.001*	1.761
Knee flexion angle (degree) over trip side at reactive step touchdown	30.38 (16.27)	18.00 (10.36)	0.05*	0.874
Ankle dorsiflexion angle (degree) over trip side at reactive step touchdown	14.41 (6.11)	7.49 (5.47)	0.01*	1.182
Shoulder abduction angle (degree) over reactive step side at reactive step touchdown	25.22 (10.54)	14.35 (6.39)	0.01*	1.198
Shoulder external rotation angle (degree) over trip side at reactive step touchdown	33.09 (19.94)	14.10 (10.78)	0.01*	1.130
Ankle dorsiflexion moment over trip side normalized by body weight at reactive step touchdown (Nm/kg)	0.27 (0.27)	0.68 (0.37)	< 0.001*	1.314
Reactive step excursion time (s)	0.49 (0.25)	0.29 (0.08)	0.02*	1.002
Reactive step length (m)	0.29 (0.18)	0.35 (0.14)	0.37	0.382
Reactive step speed (m/s)	0.62 (0.26)	1.26 (0.47)	< 0.001*	1.744

* *p* ≤ 0.05, significantly different between groups.

Large effect sizes (Cohen's *d*) were observed for trunk flexion (1.76), ankle dorsiflexion angle (1.18), ankle dorsiflexion moment (1.31), shoulder abduction (1.20), shoulder external rotation (1.13), and knee flexion angle (0.87), indicating substantial biomechanical differences of potential clinical relevance. Trunk flexion emerged as a key factor in reactive recovery post‐trip perturbation in stroke survivors (Table 3). Large effect sizes (Cohen's *d* > 0.8) were observed for these key variables. These underscore substantial and clinically meaningful biomechanical differences in reactive step quality between stroke participants and healthy controls.

Other joint angles and moments from the full‐body model were analyzed to assess differences between stroke participants and healthy controls at reactive step touchdown. No statistically significant differences were observed for these variables, suggesting that the primary distinctions in movement patterns may be localized to specific joints of the reactive step. These findings support the focus on targeted joint analysis in future studies, as broader full‐body measures did not reveal group‐level differences at this stage of the movement.

**Figure 2:**
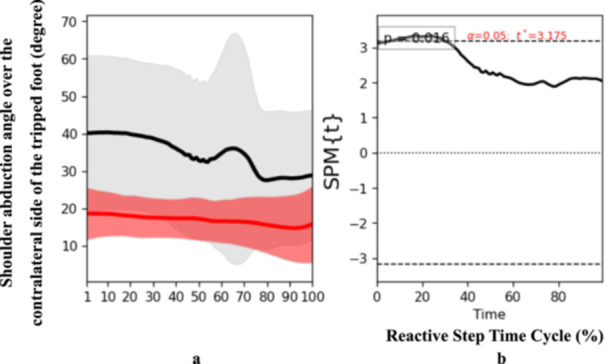
Pattern of angle of shoulder abduction over the contralateral side of the tripped foot during the reactive step of participants with stroke (gray line) and healthy controls (red line) during the simulated trip perturbation. (a) Time‐series means and standard deviations (degree) of angle of shoulder abduction over the contralateral side of the tripped foot; (b) the results of the 1D statistical parametric mapping (SPM) paired Student's *t*‐test analysis of the angle of shoulder abduction over the contralateral side of the tripped foot (degree).

**Figure 3:**
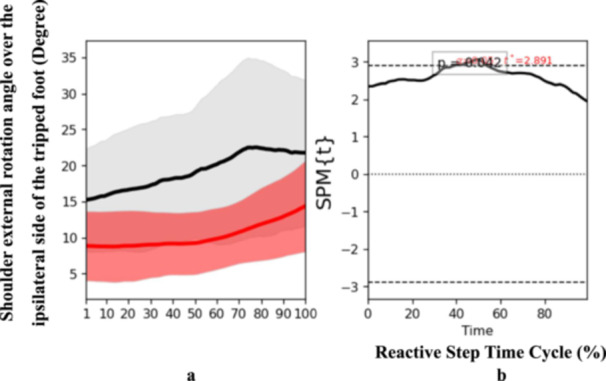
Pattern of angle of shoulder external rotation over the ipsilateral side of the tripped foot during the reactive step of participants with stroke (gray line) and healthy controls (red line) during the simulated trip perturbation. (a) Time‐series means and standard deviations (degree) of angle of shoulder external rotation over the ipsilateral side of the tripped foot; (b) the results of the 1D statistical parametric mapping (SPM) paired Student's *t*‐test analysis of the angle of shoulder external rotation over the ipsilateral side of the tripped foot (degree).

SPM1D results revealed the observed time series of joint angles and moment were not randomly derived but exhibited deterministic patterns in particular of initial phase and middle phase of the reactive step cycle including increased shoulder abduction angle over the reactive side (*t* = 3.175, *p* = 0.02) at initial phase (Figure 2), and increased shoulder external rotation angle over the perturbated side (*t* = 2.891, *p* = 0.04) at middle phase in stroke participants compared to controls (Figure 3). These findings suggest enhanced upper body recruitment as a compensatory strategy during early reactive stepping phases.

**Figure 4:**
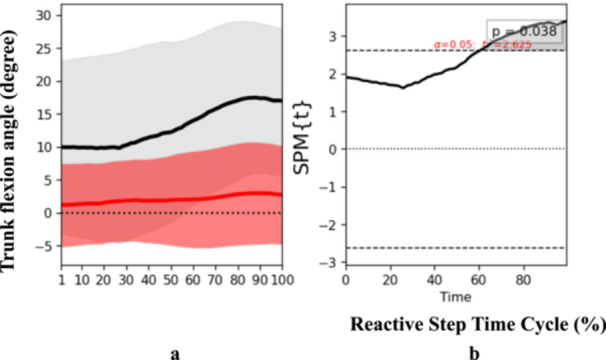
Pattern of angle of trunk flexion during the reactive step of participants with stroke (gray line) and healthy controls (red line) during the simulated trip perturbation. (a) Time‐series means and standard deviations (degree) of the angle of trunk flexion; (b) the results of the 1D statistical parametric mapping (SPM) paired Student's *t*‐test analysis of the angle of trunk flexion (degree).

**Figure 5:**
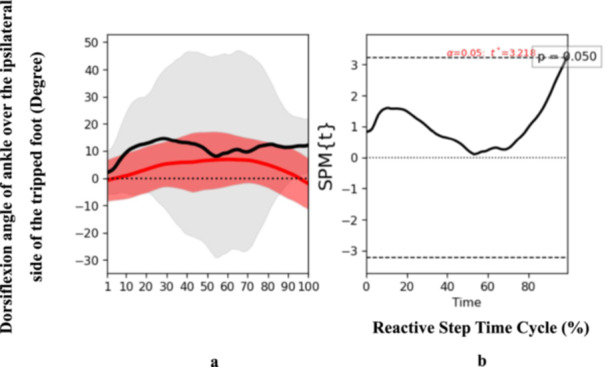
Pattern of dorsiflexion angle of ankle over the ipsilateral side of the tripped foot during the reactive step of participants with stroke (gray line) and healthy controls (red line) during the simulated trip perturbation. (a) Time‐series means and standard deviations (degree) of dorsiflexion angle of ankle over the ipsilateral side of the tripped foot; (b) the results of the 1D statistical parametric mapping (SPM) paired Student's *t*‐test analysis of the dorsiflexion angle of ankle over the ipsilateral side of the tripped foot (degree).

**Figure 6:**
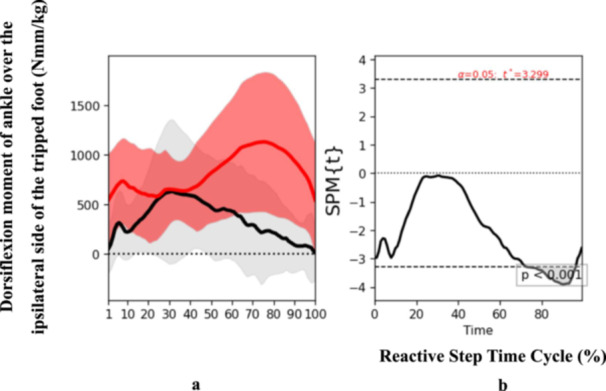
Pattern of dorsiflexion moment of ankle over the ipsilateral side of the tripped foot patterns during the reactive step of participants with stroke (gray line) and healthy controls (red line) during the simulated trip perturbation. (a) Time‐series means and standard deviations of dorsiflexion moment of ankle over the ipsilateral side of the tripped foot; (b) the results of the 1D statistical parametric mapping (SPM) paired Student's *t*‐test analysis of the dorsiflexion moment of ankle over the ipsilateral side of the tripped foot.

**Figure 7:**
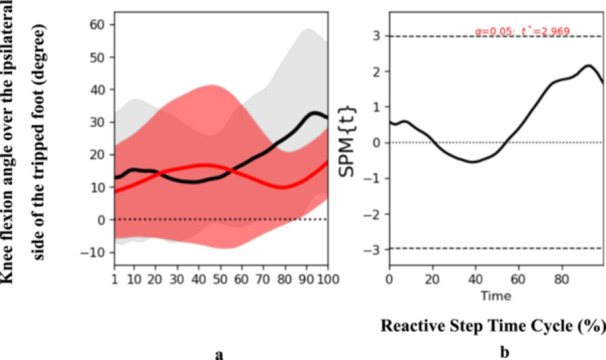
Pattern of angle of knee flexion over the ipsilateral side of the tripped foot during the reactive step of participants with stroke (gray line) and healthy controls (red line) during the simulated trip perturbation. (a) Time‐series means and standard deviations (degree) of angle of knee flexion over the ipsilateral side of the tripped foot; (b) the results of the 1D statistical parametric mapping (SPM) paired Student's *t*‐test analysis of the angle of knee flexion over the ipsilateral side of the tripped foot (degree).

At the end phase, specific biomechanical characteristics were also determined. Participants who had experienced a stroke presented an increased trunk flexion angle (*t* = 2.625, *p* = 0.04) at the end phase of the reactive step cycle (Figure 4), suggesting an altered strategy to regain balance and prevent a fall post‐perturbation. Additionally, ankle dorsiflexion angle over the trip side also exhibited significant discrepancies (*t* = 3.218, *p* = 0.05) at the end phase (Figure 5). Notably, there was a reduction in the moment of ankle dorsiflexion over the trip side among the stroke participants (*t* = 3.299, *p* < 0.001) (Figure 6), implying a potential weakness or coordination deficit that could contribute to fall risk. Contrary to expectations, knee flexion angle on the trip side did not differ significantly between groups across the reactive step cycle (Figure 7).

## 4 Discussion

Previous studies had provided valuable insights into the biomechanical factors underlying fall risk in stroke survivors [5, 6, 7]. However, most earlier studies prioritized biomechanical analysis during the static or standing trip perturbations, which limits the direct applicability to more dynamic and functional scenarios such as walking with unexpected trips. In addition, there is a notable paucity of research investigating phase‐specific biomechanical adaptations throughout the entirety of reactive stepping responses during gait perturbations. These methodological issues result in a limited understanding of the temporal and segmental coordination required for successful balance recovery in real‐world contexts. In addition, it highlights the need for studies employing gait‐based perturbation paradigms that capture the full complexity of post‐stroke reactive locomotor control.

Given that impaired reactive stepping is a leading cause of falls and subsequent morbidity in stroke survivors, results obtained from this study contribute through enhancing the understanding of trunk, limb, and upper limb adaptations, as well as their implications for balance reactive step, which help inform the development of more targeted and effective physiotherapy assessments and interventions.

The observed biomechanical differences between stroke participants and healthy controls during reactive stepping following simulated trip perturbations highlight the complex motor adaptations and stability challenges post‐stroke. This study successfully captured joint movement patterns throughout the entire reactive step, demonstrating that the technology‐assisted simulated trip perturbation by the SBT method is a reliable and safe tool for evaluating biomechanical responses during reactive stepping in stroke participants.

Stroke participants exhibited significantly slower self‐selected usual walking speeds (0.27 ± 0.10 m/s) and altered joint angles, reflecting compensatory mechanisms aimed at maintaining balance and stability. This walking speed aligns with classifications of slow gait in prior post‐stroke SBT literature [18]. Research examining brain activation patterns post‐stroke indicates increased contralesional activity in motor execution and sensory integration areas, particularly pronounced during slower, cautious walking [19]. Slower walking speeds are associated with less automatic gait control, whereas faster walking correlates with brain adaptations necessary for navigating complex environments [20]. Consistent with this, our data suggest that slow self‐selected walking speeds reflect cautious gait strategies and diminished automaticity in stroke survivors. The observed slower walking speeds and altered joint kinematics in stroke participants reflect a fundamental reduction in the automaticity of gait control. This less automatic control may result in greater reliance on conscious, cortical processes to maintain balance and coordination during walking, increasing cognitive load and reducing gait adaptability.

Biomechanical responses to perturbations in stroke survivors are influenced not only by walking speed but also by neuromuscular deficits and compensations, resulting in amplified biomechanical differences despite slower speeds [21]. Besides, stroke participants showed a significantly slower reactive step recovery time, approximately double that of controls, even when the reactive step was performed with the nonparalyzed (nonaffected) limb. This finding highlights the importance of considering the nonparalyzed limb's role in rehabilitation.

Slower reactive stepping speeds in individuals after stroke are likely driven by deficits across multiple neural control processes. Recent neurophysiological research demonstrates that stroke survivors exhibit delayed cortical processing in response to balance perturbations, as shown by slower evoked cortical responses (N1 potentials) during reactive balance tasks [22]. This delay in cortical activation impairs rapid error detection and motor planning, limiting the ability to generate timely postural adjustments during sudden balance challenges. The deficit is especially pronounced when the paretic limb is involved, as its motor capacity is further limited by the stroke lesion. Biomechanically, these neural control impairments manifest as delayed step initiation, shorter and less effective compensatory steps, and reduced capacity to generate stabilizing limb forces together resulting in slower and less efficient reactive stepping [23]. Sensory transmission and integration are also disrupted post‐stroke, further contributing to delays and deficits in step execution. Taken together, these findings support that stroke‐related disruptions in both cortical and subcortical neural control mechanisms are key contributors to slower and less effective reactive stepping, increasing fall risk during daily activities. Our results highlight the importance of rehabilitation strategies targeting both the speed and automaticity of neural responses for optimal balance recovery.

The hypotheses were accepted, demonstrating that statistically significant differences existed between stroke participants and healthy controls in the overall biomechanical characteristics of reactive stepping and the joint angular profiles during reactive stepping, identifying distinct difference‐in‐difference patterns across the movement continuum.

Stroke participants exhibited increased shoulder abduction and external rotation angles during the reactive step, indicating a greater reliance on upper limb strategies to maintain postural control and stability. This aligns with evidence from healthy adults, where arm movements play a crucial role in managing the body's center of mass (COM) excursion to prevent balance loss during trip perturbations [24]. Similarly, deviations in trunk angle during external perturbations have been documented in slip studies [25, 26]. Employing upper limb strategies likely helps counteract COM excursion by adjusting body orientation toward a more stable posture, reducing fall risk [27]. Our findings extend this understanding to stroke survivors, highlighting the potential therapeutic value of targeting upper limb movements during rehabilitation.

Significant differences were observed between stroke and control groups in trunk flexion angle and various joint angles at reactive step touchdown, with large effect sizes (Cohen's *d*). These substantial effects emphasize the clinical relevance of altered biomechanical movement patterns post‐stroke. The pronounced trunk flexion plays a critical role during the dynamic task of trip recovery, likely influencing performance and injury risk. Large deviations in joint angles, especially in upper and lower extremities, underscore the necessity of refined rehabilitation protocols focused on these biomechanical parameters to improve dynamic stability and functional outcomes.

SPM1D analysis revealed distinct compensatory upper limb biomechanics in stroke survivors, particularly involving the shoulder region during the early phase of the reactive step. These biomechanical strategies likely contribute to force redistribution throughout the body, promoting postural stability and reducing fall risk. Recent research highlights that arm movements, particularly at the shoulder joint, play a vital role in stabilizing COM during balance recovery following perturbations. In response to a trip or slip, individuals rapidly engage shoulder abduction and external rotation to move the arms outward or upward. This movement serves two primary biomechanical functions, including by altering COM trajectory with the upper limbs act as dynamic counterweights, redirecting the COM toward a safer position within the base of support, thereby reducing the risk of falling [28, 29]. Besides, the increased range of motion in these shoulder movements raises the moment of inertia, which helps slow down or reverse the body's angular momentum during destabilizing events, supporting more effective postural control [30]. Understanding these mechanisms has practical implications for rehabilitation including therapies that enhance upper limb coordination, strength, and control could improve balance recovery. Incorporating upper limb movements into balance training may facilitate integration of upper and lower body control, enhancing overall postural stability. Early‐stage rehabilitation might allow unrestricted arm movement during balance exercises or involve physiotherapist‐facilitated specific shoulder motions during different reactive step phases. As recovery progresses, selectively restricting upper limb motion could increase task difficulty and further challenge balance control. Future randomized controlled trials are warranted to evaluate the efficacy of this graduated strategy for fall prevention in stroke populations.

During the end phase of the reactive step, stroke participants showed increased trunk flexion and ankle dorsiflexion angles, indicative of delayed compensatory responses. The reduced ankle moment on the perturbed side suggests muscle weakness or impaired motor control over the affected side of stroke participants, limiting the ability to generate adequate stabilizing force and thereby increasing fall risk. Unlike controls, who maintained relatively consistent trunk flexion, stroke survivors demonstrated continuous forward trunk lean, especially at the end phase of the reactive step. This increased forward trunk flexion likely serves to lower the COM and aid postural control; however, when combined with limited ankle support, it may also contribute to instability and greater fall risk.

### 4.1 Implications for Physiotherapy Practice

This study represents a novel application of SPM1D for analyzing movement trajectories and biomechanical responses to trip perturbations, specifically in stroke survivors, filling a gap in existing literature. The findings emphasize the complex interplay of compensatory mechanisms involving the trunk, lower limbs, and upper limbs post‐stroke, and highlight critical targets for comprehensive rehabilitation aimed at enhancing dynamic balance and reducing falls.

### 4.2 Limitation

Several limitations should be considered when interpreting the results of this study. First, the focus on the nonparalyzed limb during reactive stepping may not fully capture the complexity of real‐world scenarios, where trips can occur on either limb. Including both the paralyzed and nonparalyzed sides in future studies would provide a more comprehensive understanding of reactive balance control in stroke survivors. Second, the sample size was limited and consisted solely of community‐dwelling stroke participants under the age of 60. This restriction may limit the generalizability of the findings to older individuals or those at different stages of stroke recovery, who often present distinct biomechanical and functional profiles.

A further limitation is the between‐group difference in self‐selected usual walking speeds, which could have influenced biomechanical responses. Slower walking speeds have been associated with smaller COM excursions and more conservative balance strategies, potentially contributing to some of the observed differences. Future studies could consider including both self‐selected and standardized walking speed conditions to better disentangle speed‐related effects from stroke‐related impairments. Despite these limitations, this study offers valuable insights from a technology‐assisted biomechanical perspective and contributes meaningfully to current knowledge, laying important groundwork for future research.

Building on these findings, future investigations should explore interventions designed to enhance adaptive variability and target specific phases of the reactive stepping cycle. By deepening our understanding of biomechanical responses in stroke survivors and developing evidence‐based rehabilitation strategies, we can advance post‐stroke care, ultimately helping individuals regain confidence, mobility, and independence. Furthermore, there is potential to develop objective biomechanical assessment tools specifically tailored for stroke survivors that encompass a variety of real‐world scenarios. These tools would allow for precise evaluation of reactive balance and motor control under different conditions, thereby facilitating personalized rehabilitation planning and enhancing clinical outcomes by using the current design.

## 5 Conclusion

This research offers valuable insights into the movement patterns and biomechanical characteristics associated with reactive stepping in stroke participants during simulated trip perturbations on the SBT platform, using SPM1D analysis. Significant deviations in biomechanical parameters were identified, particularly during distinct phases of the entire reactive step cycle. These findings emphasize the complexity of post‐stroke motor control adaptations and highlight the challenges stroke participants face in maintaining stability during dynamic reactive tasks. By employing SPM1D alongside the SBT methodology, this study advances biomechanical analysis beyond the limitations of previous research that focused primarily on discrete, single‐time‐point measurements, providing a comprehensive temporal profile of reactive stepping biomechanics in this population.

## Author Contributions

**Suk‐Ping Chan:** conceptualization, data curation, formal analysis, investigation, methodology, project administration, writing – original draft, writing – review and editing. **Patrick Wai‐Hang Kwong:** conceptualization, formal analysis, methodology, writing – review and editing. **Gladys Lai‐Ying Cheing:** conceptualization, methodology, writing – review and editing. **Sharon Man‐Ha Tsang:** conceptualization, methodology, writing – review and editing.

## Ethics Statement

This cross‐sectional study received approval from the Research Ethics Committee (Kowloon Central/Kowloon East) and the Institutional Review Board of The Hong Kong Polytechnic University (reference number: HSEARS20210916001). The study objectives, procedural details, benefits, and potential risks were thoroughly explained to all participants. Written informed consent was obtained from each participant prior to commencing the research. Participants who met the inclusion criteria and had no exclusion criteria were fully briefed on the study procedures and protocols, and provided signed informed consent before participation.

## Conflicts of Interest

The authors declare no conflicts of interest.

## Transparency Statement

The lead author, S. P. Chan, affirms that this manuscript is an honest, accurate, and transparent account of the study being reported; that no important aspects of the study have been omitted; and that any discrepancies from the study as planned (and, if relevant, registered) have been explained.

## Data Availability

The authors confirm that the data supporting the findings of this study are available within the article.
